# More Than Just a Polyp: Diagnosis of Tuberculosis From a Screening Colonoscopy

**DOI:** 10.7759/cureus.13216

**Published:** 2021-02-08

**Authors:** Adham E Obeidat, Thomas Namiki, Traci T Murakami

**Affiliations:** 1 Department of Internal Medicine, The Queen’s Medical Center, Honolulu, USA; 2 Department of Pathology, The Queen’s Medical Center, Honolulu, USA; 3 Department of Gastroenterology, The Queen’s Medical Center, Honolulu, USA

**Keywords:** colon, colonoscopy, colon cancer, screening, tuberculosis

## Abstract

The diagnosis of tuberculosis (TB) is challenging to make due to the non-specific signs and symptoms that patients usually present with. Furthermore, the endoscopic appearance of colonic TB is not specific and can mimic other more common pathologies such as Crohn’s disease and colonic malignancy. We report the case of a 66-year-old female who presented for a routine screening colonoscopy and was diagnosed with disseminated TB after histopathological examination of the discovered polyps.

## Introduction

Tuberculosis (TB) is one of the 10 most common causes of death worldwide and the leading cause of death from a single infectious agent. A total of 1.4 million people died from TB in 2019 [1]. TB most commonly affects the lungs, but can also be extra-pulmonary [2]. *Mycobacterium tuberculosis* can infect the gastrointestinal (GI) tract, most commonly in the terminal ileum, small intestine, and colon [3-7]. GI TB is the sixth most common site of extra-pulmonary TB, and before the development of antitubercular drugs, autopsies showed that GI tract was involved in more than 90% of the cases [5].

Patients usually present with non-specific symptoms such as abdominal pain, abdominal distention, weight loss, decreased appetite, and may also have diarrhea and vomiting. Signs are non-specific as well, including malnutrition, ascites, abdominal distention, or a palpable mass [3-9]. On colonoscopy, colonic TB can present as ulcerative or nodular lesions, strictures, and, less frequently, as colon polyps [4,7,8,10]. Given the vague presentation and varied endoscopic findings of colonic TB, a high degree of clinical suspicion is needed to make this diagnosis. We present the case of a 66-year-old female who had colonic polyps on a screening colonoscopy and was diagnosed with disseminated TB after the histopathological examination of the polyps.

This case was previously presented as a poster at the American College of Gastroenterology annual scientific meeting (virtual platform) October 23-28, 2020.

## Case presentation

A 66-year-old female with past medical history significant for hypertension and goiter was referred to the GI clinic by her primary care physician for a screening colonoscopy. She had no prior colonoscopy. She was born in the Philippines and moved to the United States 20 years ago. The patient only complained of fatigue and denied loss of appetite, weight loss, abdominal pain, nausea, vomiting, change in bowel habits, or rectal bleeding. Her physical examination, including abdominal examination, was unremarkable. She had no family history of GI malignancy and denied any tobacco, alcohol, or illicit drug use.

**Figure 1:**
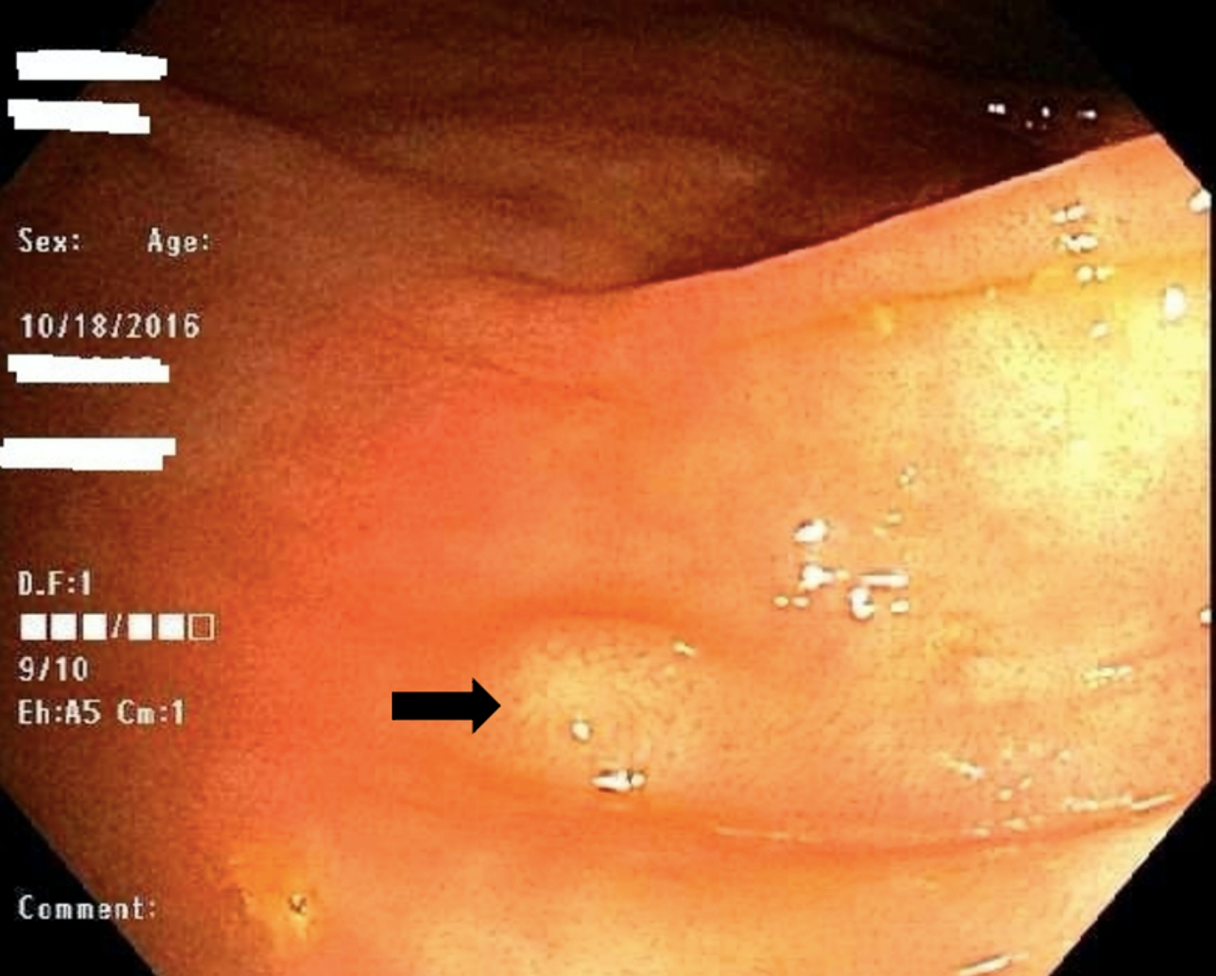
Endoscopic image showing a small sessile polyp in the ascending colon.

**Figure 2:**
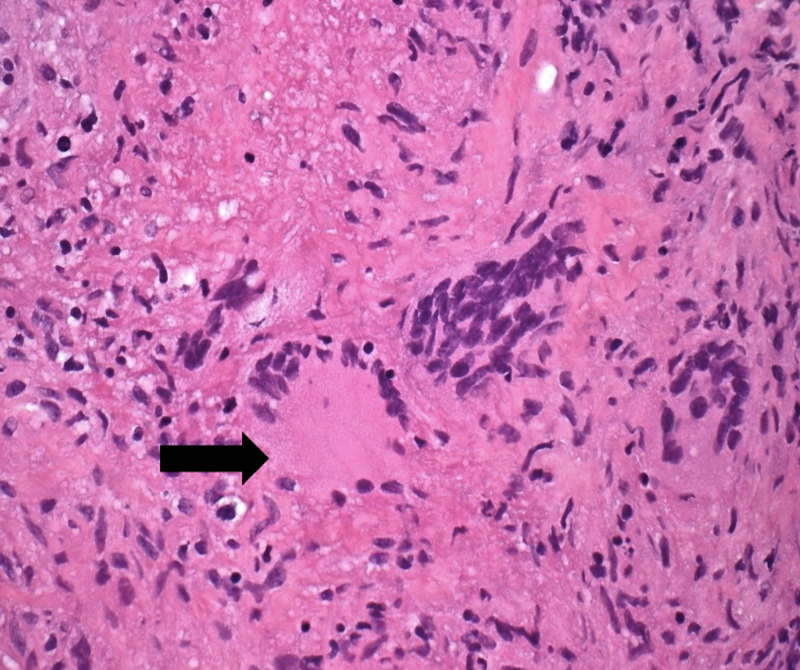
Photomicrograph of colonoscopic biopsy specimen showing granulomas with caseating necrosis.

On colonoscopy, three sessile polyps between 2 and 4 mm in size were identified in the ascending colon (Figure 1), in addition to a 5 mm hyperplastic sessile polyp in the cecum. Polyps were resected and biopsies were sent for evaluation. Histopathological evaluation of the resected polyps from ascending colon revealed mucosal granulomas with caseating necrosis (Figure 2). Acid-fast bacilli (AFB) and fungal stains were negative. Upon receiving the pathology report, a chest X-ray was performed and showed a collapsed right upper lobe associated with a central lung mass concerning for TB versus malignancy. Computed tomography (CT) scan of the chest was done which showed a 10 cm mass centered in the right upper lobe and paramediastinal region with cavitation. Moreover, nodular pleural thickening, hilar and right internal mammary adenopathy, and diffused nodular opacities were identified.

The patient was referred to the pulmonary clinic for further evaluation of the lung mass. Sputum AFB smear and culture were done and were positive for *M. tuberculosis*. The patient underwent CT-guided biopsy of the lung mass, and histopathological examination was diagnostic for pulmonary TB. She received antitubercular treatment with rifampin, isoniazid, ethambutol, and pyrazinamide for nine months, and her fatigue subsequently resolved. Repeat sputum smear and culture were negative for *M. tuberculosis*. Given the resolution of symptoms after treatment, a colonoscopy was not repeated, and a follow-up chest CT showed a regression of the lung mass size and associated adenopathy.

## Discussion

GI TB is not uncommon and remains a significant problem in immunosuppressed individuals and those born in endemic countries. While the incidence of pulmonary TB is decreasing, the incidence of extra-pulmonary TB is increasing, which may be due to the improvement in life expectancy, increased predominance of females in the population, and the decrease of BCG vaccines worldwide [11]. In the last two decades, the incidence of GI TB, including colonic TB, has been increasing, which may be attributed to the increased incidence of HIV infections and the rising use of immunosuppressive drugs [5]. One study showed that co-infection of TB with HIV can be as high as 73% [12]. TB is also a concern in patients receiving biological agents for conditions like inflammatory bowel disease and rheumatoid arthritis [7,13]. Reported studies found that 39.5% of GI TB is accompanied by another source of infection where the lung is the most common site [5].

GI TB most commonly occurs in the terminal ileum, which is attributed to the abundance of lymphoid tissue and physiological stasis allowing for more contact between the *M. tuberculosis* bacilli and the mucosa. One retrospective multi-center study showed that in 104 patients with GI TB, 44.2% had terminal ileum involvement, 34.6% had small intestine involvement, and 27.8% had colonic TB [5]. Colonic TB preferentially affects the ileocecal valve, followed by ascending, transverse, descending, and the sigmoid colon [7]. Our patient had three polyps in the ascending colon which turned out to be TB after histopathological examination.

Colonic TB presents with non-specific signs and symptoms such as abdominal pain, weight loss, decreased appetite, fever, nights sweats, diarrhea, and vomiting. The most common physical examination findings are abdominal distention, palpable mass, ascites, hepatomegaly, palpable lymph nodes, and splenomegaly [3-8,11]. This enigmatic presentation contributes to the delay in diagnosis and management. Our patient’s only symptom was fatigue without associated pulmonary or GI symptoms, and her physical examination was rather unremarkable which made for a difficult diagnosis.

Diagnosis of colonic TB can be done by colonoscopy combined with histopathologic and bacteriologic examination. Findings on colonoscopy can range from non-specific ulcerations, nodular lesions, strictures, and, less commonly, polyps [4,7,8,14,15]. These nondescript endoscopic findings make colonic TB easily mistaken with other colonic pathologies such as Crohn’s disease and malignancy leading to significant delays in the diagnosis and management as well as unnecessary surgical interventions. The histopathologic examination usually reveals caseating epithelioid granulomas which are very specific for TB [7,8,14]. One retrospective study of 50 patients with colonic TB from India reported that granulomas were seen only in 18% of cases, and caseation was not detected in any case. Because of the widespread use of colonoscopy, colonic TB may be detected earlier, and therefore, the classical features of colonic TB, including caseation, are not seen [8]. AFB has been reported in 50-100% of GI TB cases, and several studies reported that AFB could not be detected on histological examination of the biopsy [8]. Although AFB was not detected in the three sessile polyps of our patient, the histopathologic examination of the specimens along with the sputum AFB stain and culture were diagnostic for colonic TB.

In general, GI TB is very responsive to medical therapy, hence, early diagnosis and treatment can prevent unnecessary surgical interventions [15]. Patients diagnosed with GI TB should receive at least six months of antitubercular therapy, which can be extended to nine or 12 months, although no difference in effectiveness has been reported [3,6,7,16,17]. Surgical intervention is usually reserved for cases with complicated intestinal obstruction, perforation, abscess, or fistula formation. Even in cases with stricture formation, medical treatment results in significant improvement in most cases [16]. Some reported studies suggest a follow-up colonoscopy in two to three months after completing medical treatment [3]. On the other hand, given the majority of colonic TB cases that resolve with medical treatment, other studies do not recommend repeat colonoscopy if the patient is asymptomatic [7,16]. Our patient received the antitubercular regimen for nine months, with good clinical response, and hence, repeat colonoscopy was not performed.

## Conclusions

This case highlights the vague presentation of TB, which can present as a few unimpressive appearing small polyps in the colon. In an asymptomatic patient, a colonoscopy may rarely play a role in the diagnosis of TB. The subtle endoscopic findings of small and benign appearing sessile polyps in the colon require a high level of suspicion to obtain biopsies for histopathologic investigation. The pathologic findings on colonoscopy triggered a workup that surprisingly found pulmonary TB with colon involvement in this patient with no clinical symptoms of TB except fatigue. When performing a routine colonoscopy in asymptomatic screening patients, it is important to be aware of the subtle endoscopic findings that may diagnose GI TB and lead to the discovery of disseminated TB.

## References

[1] (2021). Tuberculosis. https://www.who.int/news-room/fact-sheets/detail/tuberculosis.

[2] Wang X, Yang Z, Fu Y, Zhang G, Wang X, Zhang Y, Wang X (2014). Insight to the epidemiology and risk factors of extrapulmonary tuberculosis in Tianjin, China during 2006-2011. PLoS One.

[3] Yu SM, Park JH, Kim MD (2012). A case of sigmoid colon tuberculosis mimicking colon cancer. J Korean Soc Coloproctol.

[4] Alvares JF, Devarbhavi H, Makhija P, Rao S, Kottoor R (2005). Clinical, colonoscopic, and histological profile of colonic tuberculosis in a tertiary hospital. Endoscopy.

[5] Tanoglu A, Erdem H, Friedland JS (2020). Clinicopathological profile of gastrointestinal tuberculosis: a multinational ID-IRI study. Eur J Clin Microbiol Infect Dis.

[6] Miah AR, Sharma YR, Rahman MT, Raihan A, Roy PK, Hasan M (2011). Clinicopathological profile of patients with abdominal tuberculosis. J Nepal Health Res Counc.

[7] Mukewar S, Mukewar S, Ravi R, Prasad A, Dua KS (2012). Colon tuberculosis: endoscopic features and prospective endoscopic follow-up after anti-tuberculosis treatment. Clin Transl Gastroenterol.

[8] Misra SP, Misra V, Dwivedi M, Gupta SC (1999). Colonic tuberculosis: clinical features, endoscopic appearance and management. J Gastroenterol Hepatol.

[9] Breiter JR, Hajjar JJ (1981). Segmental tuberculosis of the colon diagnosed by colonoscopy. Am J Gastroenterol.

[10] Naga MI, Okasha HH, Ismail Z, El-Fatatry M, Hassan S, Monir BE (2001). Endoscopic diagnosis of colonic tuberculosis. Gastrointest Endosc.

[11] García-Rodríguez JF, Álvarez-Díaz H, Lorenzo-García MV, Mariño-Callejo A, Fernández-Rial Á, Sesma-Sánchez P (2011). Extrapulmonary tuberculosis: epidemiology and risk factors. Enferm Infecc Microbiol Clin.

[12] Heunis JC, Wouters E, Norton WE, Engelbrecht MC, Kigozi NG, Sharma A, Ragin C (2011). Patient- and delivery-level factors related to acceptance of HIV counseling and testing services among tuberculosis patients in South Africa: a qualitative study with community health workers and program managers. Implement Sci.

[13] Singh JA, Wells GA, Christensen R (2011). Adverse effects of biologics: a network meta-analysis and Cochrane overview. Cochrane Database Syst Rev.

[14] Shibagaki K, Miyaike J, Onji M (2015). Submucosal tumor-like lesion originating from colon tuberculosis: a case report and review of the literature. Clin J Gastroenterol.

[15] Sato S, Yao K, Yao T, Schlemper RJ, Matsui T, Sakurai T, Iwashita A (2004). Colonoscopy in the diagnosis of intestinal tuberculosis in asymptomatic patients. Gastrointest Endosc.

[16] Debi U, Ravisankar V, Prasad KK, Sinha SK, Sharma AK (2014). Abdominal tuberculosis of the gastrointestinal tract: revisited. World J Gastroenterol.

[17] Balasubramanian R, Nagarajan M, Balambal R (1997). Randomised controlled clinical trial of short course chemotherapy in abdominal tuberculosis: a five-year report. Int J Tuberc Lung Dis.

